# Chronic Obstructive Pulmonary Disease and Employment Among Massachusetts Adults

**DOI:** 10.5888/pcd17.200116

**Published:** 2020-11-19

**Authors:** Kathleen Fitzsimmons, Elise Pechter, Emily Sparer-Fine

**Affiliations:** 1 Occupational Health Surveillance Program, Bureau of Community Health and Prevention, Massachusetts Department of Public Health, Boston, Massachusetts

## Abstract

Occupational exposure may cause or exacerbate chronic obstructive pulmonary disease (COPD), but employment may also enhance health and well-being for people with the disorder. We used self-reported data from the 2011–2017 Massachusetts Behavioral Risk Factor Surveillance System to examine COPD and employment among adults aged 40 to 70. Thirty-nine percent of adults with COPD were employed. Workers with COPD were more likely than those without COPD to report indicators of poor physical and mental health, and distribution by occupation differed between the 2 groups. Findings suggest workplace interventions may be needed to prevent respiratory exposures and enhance support for employees with COPD.

**Summary**

**What is already known about this topic?**

Chronic obstructive pulmonary disease (COPD) is an important cause of disability and death in the United States. Occupational exposure accounts for 10% to 15% of the total burden, but employment may also enhance health and well-being for people with COPD.

**What is added by this report?**

Massachusetts workers with COPD were more likely than those without COPD to report indicators of poor physical and mental health. Distribution by occupation differed between the two groups.

**What are the implications for public health practice?**

Findings suggest that workplace interventions that support and retain workers with COPD may be needed.

## Objective

Chronic obstructive pulmonary disease (COPD), a chronic, progressive lung disease, affects an estimated 15.5 million US adults (1). It is a leading cause of death and imposes a high human and financial toll in terms of hospital visits and related charges (2,3). Work in certain industries and occupations is associated with COPD, and workplace exposure accounts for 10% to 15% of the disease burden (4–6). However, employment may enhance health and well-being for people with COPD (7). Our objective was to examine COPD and employment among Massachusetts adults, including occupation, and compare workers with and without COPD to inform interventions that support health and the ability to work.

## Methods

The Behavioral Risk Factor Surveillance System (BRFSS) is an annual state-based, random-digit–dialed landline and cellular telephone survey of noninstitutionalized adults aged 18 or older that collects prevalence data on health conditions, health-related behaviors, and indicators of health care access (8). For our cross-sectional study, we used self-reported data from the 2011–2017 Massachusetts BRFSS. Annual survey response rates from the 2011–2017 survey ranged from 32.5% to 42.0%. The data were weighted to represent the adult population of Massachusetts. Our analysis was restricted to adults aged 40 to 70 years who answered both the employment and COPD questions (N = 56,052). COPD was defined as an affirmative response to the question, “Has a doctor, nurse, or other health professional ever told you that you had chronic obstructive pulmonary disease or COPD, emphysema, or chronic bronchitis?” We defined workers as respondents who reported being employed for wages, self-employed, or out of work for less than 1 year. Open-ended questions on occupation and industry were asked of workers each year in the Massachusetts BRFSS survey. Based on responses, 2002 Census occupation codes (COC) were assigned by the National Institute for Occupational Safety and Health by using computer-assisted methods and were included in the annual data sets. Individual codes were grouped into the following standard occupation categories for analysis: Management, Business and Financial Operations (COC 0010–0950); Professional and Related (1000–3540); Service (3600–4650); Sales and Related (4700–4960); Office and Administrative Support (5000–5930); Transportation and Material Moving (9000–9750); and Other. Weighted percentages and 95% CIs were estimated by using SAS version 9.4 (SAS Institute). We compared weighted distributions by using χ^2^ tests of independence.

## Results

Prevalence of COPD was 6.7% (95% CI, 6.4%–7.0%) among Massachusetts adults aged 40 to 70. Compared with adults without COPD, higher percentages of those with COPD were unable to work (33.3% vs 7.4%), retired (18.2% vs 12.2%), or out of work for 1 year or more (6.2% vs 3.8%) (Table 1). Among adults with COPD, 39.4% had worked in the past year, compared with 72.1% of those without COPD.

**Table 1:** Characteristics of Massachusetts Adults and Workers^a^ Aged 40 to 70, by COPD Status^b^, Massachusetts BRFSS, 2011–2017

Characteristic^b^	With COPD		Without COPD		*P* Value^e^
	No. Responding^c^	% (95% CI)^d^	No. Responding^c^	% (95% CI)^d^	
**All Adults, N = 56,052**					
**Employment status**	4,623	NA	51,429	NA	<.001
Unable to work		33.3 (31.0–35.6)		7.4 (7.0–7.7)	
Retired		18.2 (16.5–19.9)		12.2 (11.8–12.6)	
Out of work for ≥ 1 year		6.2 (4.8–7.5)		3.8 (3.6–4.1)	
Other (Student, homemaker)		2.9 (2.1–3.8)		4.6 (4.3–4.9)	
Employed, past year^a^		39.4 (37.0–41.8)		72.1 (71.5–72.6)	
**Workers, n = 35,906 **					
**Age, y**	1,514	NA	34,392	NA	<.001
40–50		28.5 (24.8–32.2)		45.2 (44.5–46.0)	
51–60		44.9 (40.9–48.9)		38.7 (37.9–39.4)	
61–70		26.6 (23.4–29.9)		16.1 (15.6–16.6)	
**Female**	1,514	58.0 (54.0–62.0)	34,392	48.6 (47.9–49.4)	<.001
**Race/ethnicity**	1,491	NA	33,905	NA	.07
White, non-Hispanic		87.2 (84.0–90.4)		82.6 (81.9–83.3)	
Hispanic		4.1 (2.4–5.7)		6.5 (6.0–6.9)	
Black, non-Hispanic		3.5 (1.6–5.5)		5.4 (5.0–5.7)	
Other, non-Hispanic		5.2 (2.9–7.5)		5.6 (5.2–6.1)	
**Education**	1,509	NA	34,310	NA	<.001
≤High school graduate		48.0 (44.0–52.1)		29.3 (28.5–30.1)	
>High school graduate		52.0 (47.9–56.0)		70.7 (69.9–71.5)	
**Asthma**					
Ever diagnosed with asthma^f^	1,503	45.2 (41.2–49.1)	34,326	11.5 (11.0–12.0)	<.001
Current asthma^g^	1,491	38.0 (34.1–41.9)	34,233	8.1 (7.6–8.5)	<.001
**Physical health**					
Fair or poor^h^	1,513	27.9 (24.5–31.4)	34,338	7.0 (6.6–7.4)	<.001
Poor, ≥15 days past month^i^	1,486	20.6 (17.5–23.8)	33,992	5.5 (5.2–5.9)	<.001
**Poor mental health, ≥15 days past month^j^**	1,483	19.9 (16.7–23.2)	33,913	6.8 (6.4–7.2)	<.001
**Influenza vaccination, past year**	1,412	48.4 (44.2–52.5)	31,911	43.9 (43.1–44.7)	.04
**Smoking status^k^**	1,476	NA	33,244	NA	<.001
Current		39.3 (35.3–43.3)		12.4 (11.9–13.0)	
Former		36.7 (32.9–40.4)		30.3 (29.5–31.0)	
Never		24.0 (20.6–27.5)		57.3 (56.5–58.1)	
**Secondhand smoke exposure at work, past week^l^**	971	18.1 (13.9–22.4)	22,942	10.0 (9.3–10.7)	<.001
Among nonsmokers	606	15.8 (10.9–20.6)	20,193	8.8 (8.1–9.4)	<.001

Abbreviations: BRFSS, Behavioral Risk Factor Surveillance System; COPD, chronic obstructive pulmonary disease; NA, not applicable.

a Employed for wages, self-employed, or out of work for less than 1 year.

b “Yes” (with COPD) or “no” (without COPD) response to the question: “Has a doctor, nurse, or other health professional ever told you that you had chronic obstructive pulmonary disease or COPD, emphysema, or chronic bronchitis?”

c Unweighted number of respondents who answered the corresponding question(s). The numbers might not total the full study group because not all respondents answered each question.

d Weighted percentage. Percentages may not add to 100% because of rounding.

e *P* value from χ^2^ test of independence comparing weighted distributions between those with and without COPD.

f “Yes” response to the question: “Have you ever been told by a doctor or other health professional that you have asthma?”

g “Yes” responses to the questions: “Have you ever been told by a doctor or other health professional that you have asthma?” and “Do you still have asthma?”

h Responded “fair” or “poor” when asked to describe his/her overall health as excellent, very good, good, fair, or poor.

i Responded 15 days or more when asked the number days in the past month that his/her physical health, which includes physical illness and injury, had not been good.

j Responded 15 days or more when asked the number of days in the past month that his/her mental health, which includes stress, depression, and problems with emotions, had not been good.

k Current = smoked at least 100 cigarettes in his/her lifetime and currently smokes either some days or every day; former = smoked at least 100 cigarettes in his/her lifetime but no longer smokes.

l Responded 1 or more hours when asked the number of hours exposed to other people’s tobacco smoke when at work in the past 7 days.

An estimated 3.8% (95% CI, 3.5%–4.1%) of workers had COPD. Workers with COPD were more likely than those without COPD to be older, female, non-Hispanic White, and have completed high school or a lower level of education (Table 1). They were more likely to report current asthma (38.0% vs 8.1%), describe their overall health as fair or poor (27.9% vs 7.0%), and report poor mental health for ≥15 days in the previous month (19.9% vs. 6.8%). Only 48.4% reported having an influenza vaccination in the past year. Workers with COPD were more likely to be current or former smokers; 24.0% had never smoked. Among nonsmokers, 15.8% of those with COPD reported exposure to secondhand smoke at work in the past week, compared with 8.8% of those without COPD.

The overall distribution by occupation differed between the 2 groups (*P* < .001) (Table 2). Workers with COPD were more likely to work in Service (19.3% vs 11.9%) and Office and Administrative Support (16.9% vs 10.5%) occupations and less likely in Management, Business and Financial Operations (12.5% vs 17.0%) and Professional and Related (22.0% vs 35.4%) occupations. The percentage in Service occupations tended to decrease with increasing age, and the percentage in Office and Administrative Support and Professional and Related occupations tended to increase, although estimates were imprecise. The distribution of workers without COPD by occupation varied less across age groups than those with COPD.

**Table 2:** Massachusetts Workers^a^ n = 35,906 Aged 40 to 70, by Occupation^b^, COPD Status^c^, and Age Group, Massachusetts BRFSS, 2011–2017

Census Occupation (Code)	Age, y^d^			Overall^e^
	40–50	51–60	61–70	
**With COPD**				
All groups, no.^f^	290	591	459	1,340
Management, Business and Financial Operations (COC 0010–0950)	10.8 (5.9–15.7)	13.9 (9.4–18.4)	12.1 (6.9–17.3)	12.5 (9.7–15.4)
Professional and Related (1000–3540)	18.1 (12.3–23.9)	23.3 (18.1–28.5)	24.0 (18.6–29.4)	22.0 (18.8–25.2)
Service (3600–4650)	26.9 (19.8–34.0)	16.1 (10.7–21.6)	16.4 (10.9–21.9)	19.3 (15.8–22.8)
Sales and Related (4700–4960)	8.4 (3.8–12.9)	7.0 (4.0–10.1)	13.7 (8.5–19.0)	9.2 (6.8–11.6)
Office and Administrative Support (5000–5930)	14.8 (8.5–21.0)	18.7 (13.8–23.6)	16.2 (10.8–21.5)	16.9 (13.8–20.1)
Transportation and Material Moving (9000–9750)	6.4 (2.2–10.6)	4.4 (2.4–6.5)	6.9 (3.4–10.4)	5.7 (3.9–7.4)
Other	14.6 (8.0–21.2)	16.5 (10.9–22.0)	10.6 (6.5–14.7)	14.4 (11.0–17.7)
**Without COPD**				
All groups, no.^f^	10,865	12,228	6,722	29,815
Management, Business and Financial Operations (COC 0010–0950)	17.8 (16.8–18.8)	16.4 (15.5–17.4)	16.0 (14.8–17.2)	17.0 (16.4–17.6)
Professional and Related (1000–3540)	34.6 (33.4–35.9)	35.5 (34.3–36.7)	37.4 (35.8–39.0)	35.4 (34.7–36.2)
Service (3600–4650)	12.7 (11.8–13.7)	11.4 (10.6–12.3)	10.9 (9.8–12.1)	11.9 (11.4–12.5)
Sales and Related (4700–4960)	7.7 (7.0–8.4)	7.8 (7.1–8.4)	9.3 (8.3–10.4)	8.0 (7.6–8.4)
Office and Administrative Support (5000–5930)	9.6 (8.8–10.4)	11.1 (10.3–11.9)	11.3 (10.2–12.4)	10.5 (10.0–11.0)
Transportation and Material Moving (9000–9750)	3.8 (3.2–4.4)	4.2 (3.6–4.8)	3.6 (2.8–4.3)	3.9 (3.5–4.3)
Other	13.7 (12.7–14.7)	13.5 (12.5–14.5)	11.4 (10.1–12.8)	13.3 (12.6–13.9)

Abbreviations: BRFSS, Behavioral Risk Factor Surveillance System; COPD, chronic obstructive pulmonary disease.

a Employed for wages, self-employed, or out of work for less than 1 year.

b Response to the following question categorized using 2002 Census Occupation Codes: “What kind of work did you do? For example, registered nurse, janitor, cashier, auto mechanic.”

c “Yes” (with COPD) or “no” (without COPD) response to the question: “Has a doctor, nurse, or other health professional ever told you that you had chronic obstructive pulmonary disease or COPD, emphysema, or chronic bronchitis?”

d Values are percentage (95% CI) unless otherwise indicated. Percentages are weighted and may not total 100% because of rounding.

e *P* < .001 from the χ^2^ test of independence comparing the occupation distribution overall between those with and without COPD.

f Unweighted number of respondents who answered the corresponding question(s). Those with missing or noncodable responses to the occupation question (n = 4,751) were excluded from analyses of occupation group.

## Discussion

Our study quantified the relationship between COPD and employment, raising questions about how occupation-related factors may improve health and prolong careers of workers with COPD. Nearly 40% of workers in our study with COPD continued to work, despite facing challenges. Continuing employment may confer health advantages, beyond income, including health insurance–related benefits (eg, influenza vaccinations, smoking cessation programs) and psychosocial support (7). A healthy workplace, devoid of secondhand smoke, dusts, fumes, gases, and vapors may prevent COPD onset and exacerbations (6,9).

Poor physical or mental health among workers with COPD, further affected by comorbidities like asthma, may affect their capacity to work or prompt a job change (10). Continued employment may be dependent on job type, demands, and flexibility of the employer (11). Findings by occupation suggest differences in the distribution by age among workers with COPD that are not seen in those without. This may indicate a shift to jobs that are less hazardous or labor intensive or that enable disease management (eg, flexible work schedule). It may also indicate that workers in certain high-risk occupations leave the workforce at younger ages as exposures become less tolerable or as their disease progresses (11).

Our study had limitations. First, the COPD measure was based on self-report and not medically validated. However, previous research found self-report to be consistent with objective evidence of COPD (12). Next, the proportion of workers with COPD may have been underestimated because of underdiagnosis, especially in nonsmokers. Next, the Massachusetts BRFSS is limited to noninstitutionalized adults who speak English, Spanish, or Portuguese. Lastly, we cannot infer causality about COPD and employment because the BRFSS is cross-sectional.

In conclusion, our findings suggest that interventions that support and retain workers with COPD may be needed. Further research into workplace conditions and organizational factors that best promote respiratory health would inform efforts.
